# Deep Learning of Liver Contrast-Enhanced Ultrasound to Predict Microvascular Invasion and Prognosis in Hepatocellular Carcinoma

**DOI:** 10.3389/fonc.2022.878061

**Published:** 2022-07-07

**Authors:** Yafang Zhang, Qingyue Wei, Yini Huang, Zhao Yao, Cuiju Yan, Xuebin Zou, Jing Han, Qing Li, Rushuang Mao, Ying Liao, Lan Cao, Min Lin, Xiaoshuang Zhou, Xiaofeng Tang, Yixin Hu, Lingling Li, Yuanyuan Wang, Jinhua Yu, Jianhua Zhou

**Affiliations:** ^1^ Department of Ultrasound, Sun Yat-Sen University Cancer Center, State Key Laboratory of Oncology in South China, Collaborative Innovation Center for Cancer Medicine, Guangzhou, China; ^2^ School of Information Science and Technology, Fudan University, Shanghai, China

**Keywords:** deep learning, contrast-enhanced ultrasound, microvascular invasion, hepatocellular carcinoma, prognosis

## Abstract

**Background and Aims:**

Microvascular invasion (MVI) is a well-known risk factor for poor prognosis in hepatocellular carcinoma (HCC). This study aimed to develop a deep convolutional neural network (DCNN) model based on contrast-enhanced ultrasound (CEUS) to predict MVI, and thus to predict prognosis in patients with HCC.

**Methods:**

A total of 436 patients with surgically resected HCC who underwent preoperative CEUS were retrospectively enrolled. Patients were divided into training (*n* = 301), validation (*n* = 102), and test (*n* = 33) sets. A clinical model (Clinical model), a CEUS video-based DCNN model (CEUS-DCNN model), and a fusion model based on CEUS video and clinical variables (CECL-DCNN model) were built to predict MVI. Survival analysis was used to evaluate the clinical performance of the predicted MVI.

**Results:**

Compared with the Clinical model, the CEUS-DCNN model exhibited similar sensitivity, but higher specificity (71.4% vs. 38.1%, *p* = 0.03) in the test group. The CECL-DCNN model showed significantly higher specificity (81.0% vs. 38.1%, *p* = 0.005) and accuracy (78.8% vs. 51.5%, *p* = 0.009) than the Clinical model, with an AUC of 0.865. The Clinical predicted MVI could not significantly distinguish OS or RFS (both *p* > 0.05), while the CEUS-DCNN predicted MVI could only predict the earlier recurrence (hazard ratio [HR] with 95% confidence interval [CI 2.92 [1.1–7.75], *p* = 0.024). However, the CECL-DCNN predicted MVI was a significant prognostic factor for both OS (HR with 95% CI: 6.03 [1.7–21.39], *p* = 0.009) and RFS (HR with 95% CI: 3.3 [1.23–8.91], *p* = 0.011) in the test group.

**Conclusions:**

The proposed CECL-DCNN model based on preoperative CEUS video can serve as a noninvasive tool to predict MVI status in HCC, thereby predicting poor prognosis.

## Introduction

Hepatocellular carcinoma (HCC) is the sixth most common malignancy worldwide and the second leading cause of cancer-related death in China (1). For small tumors less than 5 cm, surgical resection is considered the first-line treatment. However, approximately 50% of patients suffer early recurrence within 2 years after curative hepatectomy (2).

Microvascular invasion (MVI) is a well-known risk factor for early recurrence and poor survival (3). Several studies reported that accurate preoperative prediction of MVI status may help determine surgical resection margins to improve prognosis (4, 5). Patients with MVI-positive HCC can benefit from adjuvant trans-arterial chemoembolization (TACE) (6, 7). Therefore, preoperative prediction of MVI is of great importance for effective treatment and subsequent improvement of prognosis.

However, preoperative prediction of MVI is still challenging because it can only be obtained through histopathologic examinations of the surgical resected specimens. Several studies developed clinical models based on some clinical risk factors including tumor number, size, and alpha-fetoprotein (AFP) to predict MVI status (AUC not exceeding 0.81) (8–10). Recent studies found that predictive models performed better when incorporating some radiomic features on computed tomography (CT) or magnetic resonance (MR) (11–13). However, CT or MR is performed according to a predetermined timing regime and gets a static image, which might miss typical diagnostic enhancing patterns in early or late arterial phase due to the mistiming of the arterial phase image acquisition (14).

Compared with enhanced CT and MR, contrast-enhanced ultrasound (CEUS), which allows real-time monitoring of blood perfusion of liver lesions, provides higher specificity for diagnosing HCC (15, 16). Additionally, CEUS is a favorable technique that can visualize small vascular beds during the arterial phase (17), which can be recorded as a continuous video. Hence, CEUS video may contain information regarding tumor biological behavior. However, CEUS is a video that dynamically changes with contrast injection and has a high spatial and temporal complexity. Therefore, the quantitative assessment of CEUS is difficult.

Recently, deep learning has demonstrated superior performance in dynamic video recognition and classification (18), and they provide a promising solution to quantitative assessment of CEUS video. In this study, we proposed a deep learning model based on CEUS video to predict MVI and evaluated the prognostic value of the predicted MVI.

## Materials and Methods

### Patients

**Figure 1:**
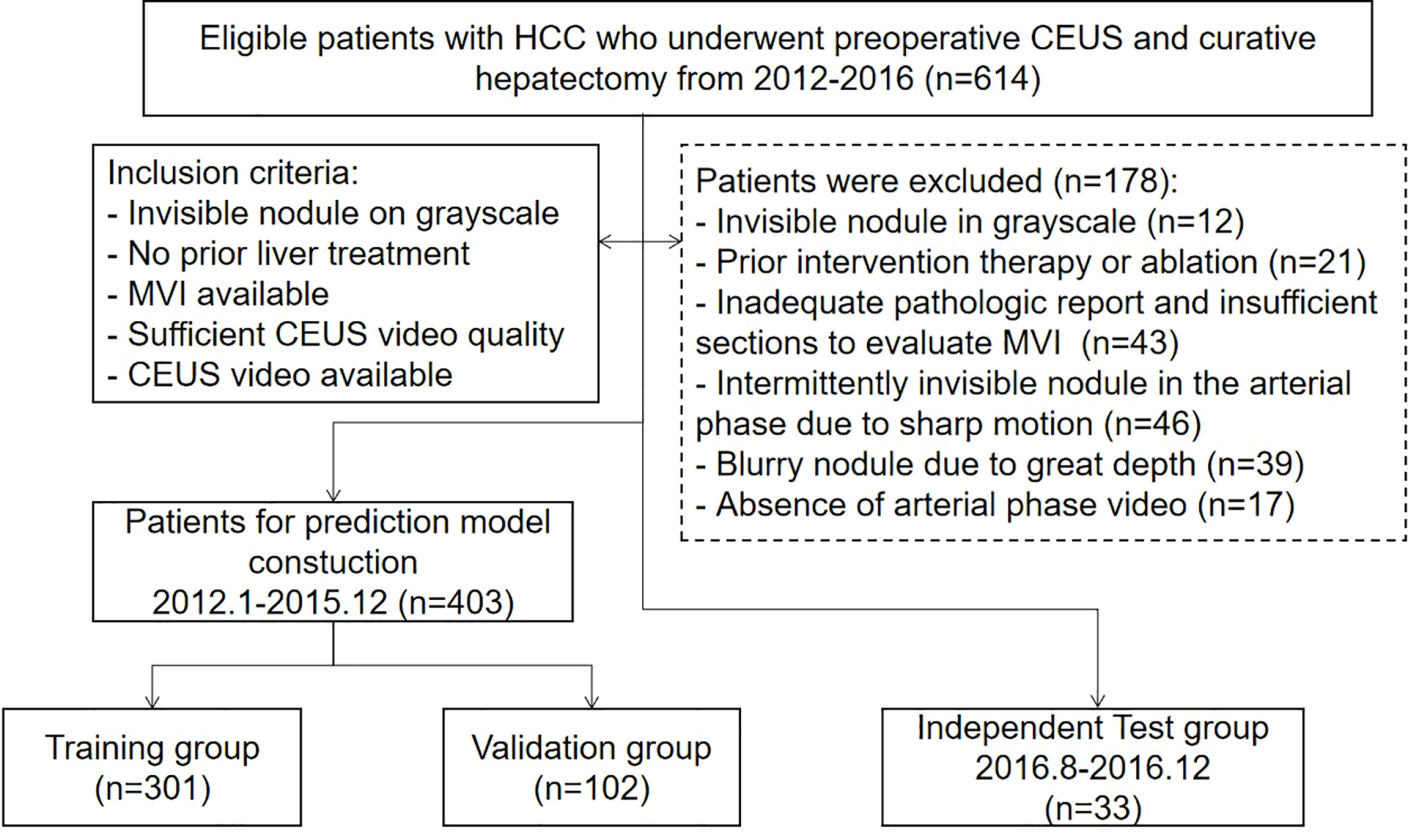
The flowchart of study group enrollment.

The institutional ethics review board approved this single-center retrospective study and waived the requirement for written informed consent. An institutional database was searched for all patients with pathologically proven HCC who underwent preoperative CEUS during two periods, January 2012 to December 2015 and August 2016 to December 2016, and found 614 patients. The final cohort was made up of 436 patients who met the following inclusion criteria (**Figure 1**): (a) nodules visible on the grayscale; (b) no previous liver cancer treatment; (c) MVI status is available in pathologic reports or sections; (d) CEUS quality appropriate to analyze; and (e) CEUS arterial phase video available. The cohort was divided into a training group (*n* = 301) and a validation group (*n* = 102) from January 2012 to December 2015 according to a ratio of 3:1, and a time-independent test group (*n* = 33) from August 2016 to December 2016.

### Clinical Parameters and Histopathological Diagnosis

The presence of MVI was mainly determined from pathologic reports and re-checked by one senior pathologist with 10-year experience. MVI was defined as the presence of tumor emboli within the vessels adjacent to HCC. Tumor maximum diameter, AFP level, and the number of nodules were recorded. The tumor maximum diameter was categorized as ≤33 mm, 33–40 mm, 40–50 mm, 50–60 mm, and >60 mm. AFP level was divided into ≤20 ng/ml, 20–400 ng/ml, and >400 ng/ml. The number of nodules was categorized as single and multiple.

### Follow-Up Surveillance

Patients were followed up regularly after surgery at intervals of 3 to 6 months, based on AFP and imaging studies. If patients were unable to visit the clinic, they were consistently kept in touch through the telephone.

### CEUS Examinations

CEUS examination was performed on an Acuson Sequoia 512 (Siemens Medical Solutions, Mountain View, CA) US system with a 4C1 convex array probe. After identifying the target lesion on the grayscale, the contrast pulse sequencing imaging mode (mechanical index, 0.19) was transferred. At the start of the CEUS mode, a volume of 2.0 ml of SonoVue (Bracco Imaging, Milan, Italy) was injected into the antecubital vein followed by a volume of 5.0 ml of saline flush. The target lesion was continuously scanned for at least 1 min immediately after the administration of the contrast agent to collect the continuous dynamic images of the arterial phase and partial portal phase. Then, the transducer ran over the entire liver and returned to the target lesion at an interval of 20–30 s until 5 min to capture the delay phase. Contrast clips were stored as video sequences or still images in Dicom format.

### Annotation and ROI Extraction of CEUS Video

Tumor segmentation was performed with ITK-SNAP (http://www.itksnap.org/) by a radiologist with 2 years of experience in CEUS, and then revised by a senior radiologist with over 20 years of CEUS experience. The tumor boundary was drawn manually on the 1-min CEUS video, and 2- and 3-min still images. Since each CEUS video consists of 430–520 frames, the workload would be tremendous for the radiologist to annotate each frame. Thus, annotations were given discontinuously and about 50–70 frames in each video have segmentations, covering all tumor moving trajectory due to patient’s breath during the CEUS examination. According to the union of given annotations in 1-min CEUS, a bounding box was extracted and extended outward by 1/4 length of each side in every video as the region of interest (ROI), as shown in **Supplementary Figure 1**.

### Developing a Clinical Parameter-Based Model

Age, sex, AFP level, tumor maximum diameter, and the number of nodules were potential clinical parameters to predict the MVI status (8–10). Univariate and multivariate logistic regression analyses were performed in the combination of the training and validation group. The parameters significant with *p* < 0.05 in the univariate analysis were taken in the multivariate analysis. The independent significant parameters made up the clinical parameter-based model (Clinical model) and participated in the later clinical parameter combining CEUS video deep learning model (CECL-DCNN model). The predictive performance of the Clinical model was evaluated in the test group.

### Developing a CEUS Video-Based Deep Learning Model

**Figure 2:**
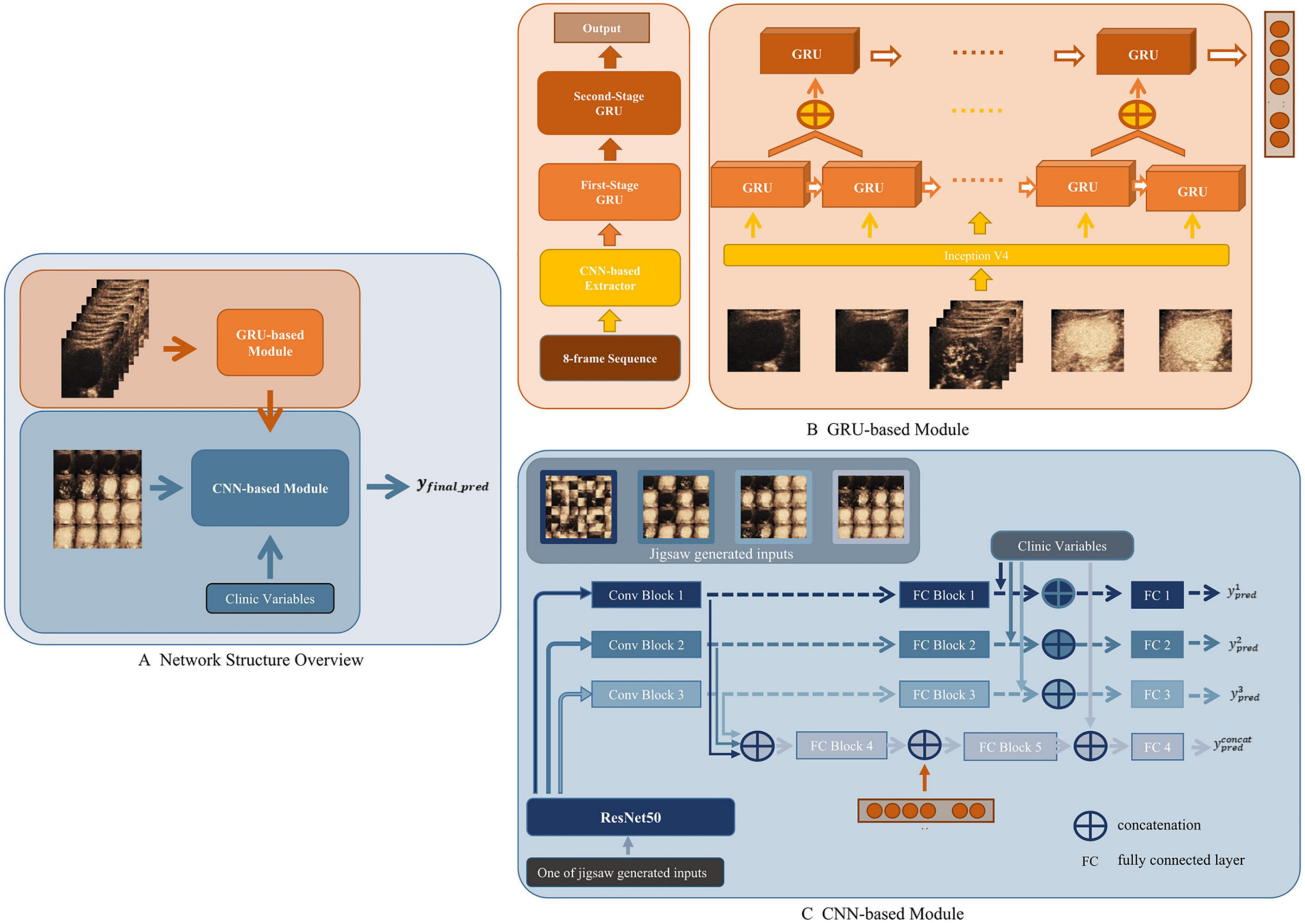
Workflow of deep convolution neural network (DCNN) analysis. **(A)** Network Structure Overview. Eight-frame sequence was the input of the Gated Recurrent Unit (GRU)-based module. Sixteen-frame spliced image, output of the GRU-based module, and the clinic variables were the inputs of the convolution neural network (CNN)-based module. **(B)** GRU-based module. The feature extracted by the CNN-based Extractor was fed into a two-stage cascade GRU to get a one-dimension output. **(C)** CNN-based module. In the training stage, a jigsaw puzzle generator was applied to randomly generate three different patch sizes of image inputs based on the 16-frame spliced image. Three generated image inputs and the original image were then fed into pipelines composed by Conv Blocks and fully connected (FC) Blocks, respectively.

A CEUS-based DCNN model (CEUS-DCNN model) was constructed to predict the MVI status. We developed a deep learning model that considered both temporal and spatial features. The whole network was divided into two parts, a Gated Recurrent Unit (GRU)-based module for extracting temporal features of contrast perfusion and a Convolution neural network (CNN)-based module for extracting spatial distribution features of contrast agents. The overall structure of the proposed model is shown in **Figure 2A**.

The GRU-based module was seamlessly composed by Inception V4 as the backbone and two-stage cascade Bidirectional GRU (19). The input of the GRU-based module was the 8-frame sequence uniformly extracted from a 1-min CEUS video. Each ROI was shrunk or enlarged to the same size, in order to be spliced into the input. An ImageNet pre-trained Inception V4 was used to extract the most significant feature of each frame—brightness. Then, the results were delivered to the first-stage GRU. As shown in **Figure 2B**, the second-stage GRU took the concatenation of adjacent outputs from the first stage as the input. The two-stage GRU extracted information about the changes in contrast brightness at different time intervals. When all frames were passed through the network, the temporal features of the whole CEUS video were obtained.

The CNN-based module was developed based on ResNet50 (20). The input of the CNN-based module was the 16-frame spliced image uniformly extracted from the 1-min CEUS video. As a backbone feature extractor, ResNet50 could produce intermediate features with a different number of channels, widths, and lengths of the feature map. In the proposed module, we used three different scales of extracted feature maps from ResNet50. As shown in **Figure 2C**, these selected feature maps on three different scales would then be fed into a similar pipeline separately to get a prediction based on its input. Specifically, there were three pipelines in the presented module. In each pipeline, features were first fed into Conv Blocks and then delivered to fully connected (FC) Blocks. The output of an FC Block would become the prediction of each pipeline. A saliency map was used to help visually explain the feature extractions (21).

As a network with fusion of temporal and spatial features, this deep learning model reasonably fused a GRU-based module and a CNN-based module, and solved the training problem of the fusion network by a progressive training strategy, which was inspired by the Progressive Multi-Granularity (PMG) training framework (22). The specific approach was to input the temporal features extracted by the GRU-based module into the FC Block of the CNN-based module (**Figure 2C**).

### Developing a CEUS Combining a Clinical Parameter-Based DCNN Model

Incorporating significant clinical parameters, we proposed the CECL-DCNN model based on the CEUS-DCNN model. Clinical parameters were concatenated with the output of the FC Blocks or the two-stage fully connected layers (**Figure 2C**). Another fully connected layer was added in each stage. The fused feature was fed into such a layer to obtain the prediction for each stage. Clinical parameters, the GRU-based module, and prediction of the stage using features obtained in the three pipelines made up the final prediction of the combined model. The detailed calculation method and training strategy are described in **Supplementary Materials**—Network Architecture.

### Statistical Analysis

Analysis of variance (ANOVA, for continuous variables) and the Mann–Whitney rank-sum test (for categorical variables) were used to compare the basic characteristics among the training, validation, and test groups. The area under the receiver operating characteristic curve (AUC) was used to quantify the discriminative efficacy for MVI prediction. The DeLong test was performed to compare the AUCs of different models. Comparisons of the sensitivity, specificity, and accuracy were performed using the chi-square test. Recurrence-free survival (RFS) and overall survival (OS) were defined as the interval between diagnosis and radiographic detection of recurrence, last follow-up, or death. Survival curves were generated with the Kaplan–Meier method and compared by a two-sided log-rank test according to the predicted MVI.

CEUS-DCNN and CECL-DCNN model building and evaluation were conducted using Python (version 2.7, https://www.python.org/). Statistical analyses were performed using a statistical software package (SPSS version 26, SPSS, Inc., Chicago, IL). Differences of *p* < 0.05 were considered statistically significant.

## Results

### Patient Characteristics

The clinical characteristics of the patients in the training, validation, and test groups are listed in **Table 1**. There was no significant difference in characteristics among the three cohorts. A total of 103 patients (34.2%) in the training cohort, 35 patients (34.3%) in the validation cohort, and 12 patients (36.4%) in the test cohort were pathologically identified with MVI.

**Table 1:** The clinical characteristics of training, validation, and test groups.

	Training (*n* = 301)	Validation (*n* = 102)	Test (*n* = 33)	*p*
Age, years	51 ± 11	52 ± 13	55 ± 11	0.144
Sex				
Male	254 (84.4%)	82 (80.4%)	27 (79.4%)	
Female	47 (15.6)	20 (19.6%)	7 (20.6%)	
AFP				0.329
≤20 ng/ml	129 (42.9%)	38 (37.3%)	16 (47.1%)	
20–400 ng/ml	77 (25.6%)	35 (34.4%)	10 (29.4%)	
>400 ng/ml	95 (31.6%)	29 (28.4%)	8 (23.5%)	
Tumor Maximum Diameter				0.969
≤33 mm	125 (41.5%)	42 (41.2%)	14 (41.2%)	
33–40 mm	48 (15.9%)	18 (17.6%)	6 (17.6%)	
40–50 mm	40 (13.3%)	10 (9.8%)	4 (11.8%)	
50–60mm	31 (10.3%)	9 (8.8%)	2 (5.9%)	
>60 mm	57 (18.9%)	23 (22.5%)	8 (23.5%)	
Number of nodules				0.940
Single	269 (89.4%)	90 (88.2%)	30 (88.2%)	
Multiple	32 (10.6%)	12 (11.8%)	4 (11.8%)	
MVI				0.992
Positive	103 (34.2%)	35 (34.3%)	12 (35.3%)	
Negative	198 (65.8%)	67 (65.7%)	22 (64.7%)	

AFP, alpha-fetoprotein; MVI, microvascular invasion.

### Performance of the MVI Prediction Model

All clinical variables were obtained preoperatively. In the combination of training and validation groups, AFP level, tumor maximum diameter, and the number of nodules were significantly associated with MVI. These three important clinical variables made up the Clinical model. The detailed results of univariate and multivariate logistic analysis are presented in **Table 2**. The performance of the Clinical model was evaluated in the test group.

**Table 2:** Univariate and multivariate logistic analysis of MVI based on clinical variable.

Variable	Univariate		Multivariate	
	OR (95% CI)	*p*	OR (95% CI)	*p*
Age, years	0.99 (0.97–1.00)	0.100	——	——
Sex, female vs. male	0.72 (0.41–1.29)	0.268	——	——
AFP, ng/ml				
20–400 vs. ≤20	1.43 (0.84–2.45)	0.186	1.62 (0.89–2.95)	0.112
>400 vs. ≤20	2.97 (1.73–5.10)	<0.001	2.76 (1.50–5.10)	0.001
Tumor Maximum Diameter, mm				
33–40 vs. ≤33	1.66 (0.83–3.33)	0.177	1.65 (0.80–3.39)	0.117
40–50 vs. ≤33	5.19 (2.60–10.35)	<0.001	4.69 (2.30–9.56)	<0.001
50–60 vs. ≤33	3.11 (1.45–6.67)	0.004	3.14 (1.44–6.84)	0.004
>60 vs. ≤33	11.41 (6.09–21.36)	<0.001	10.43 (5.43–20.05)	<0.001
Nodule number, single vs. multiple	4.43 (2.29–8.60)	<0.001	2.74 (1.30–5.79)	0.008

AFP, alpha-fetoprotein; OR, odds ratio.

**Figure 3:**
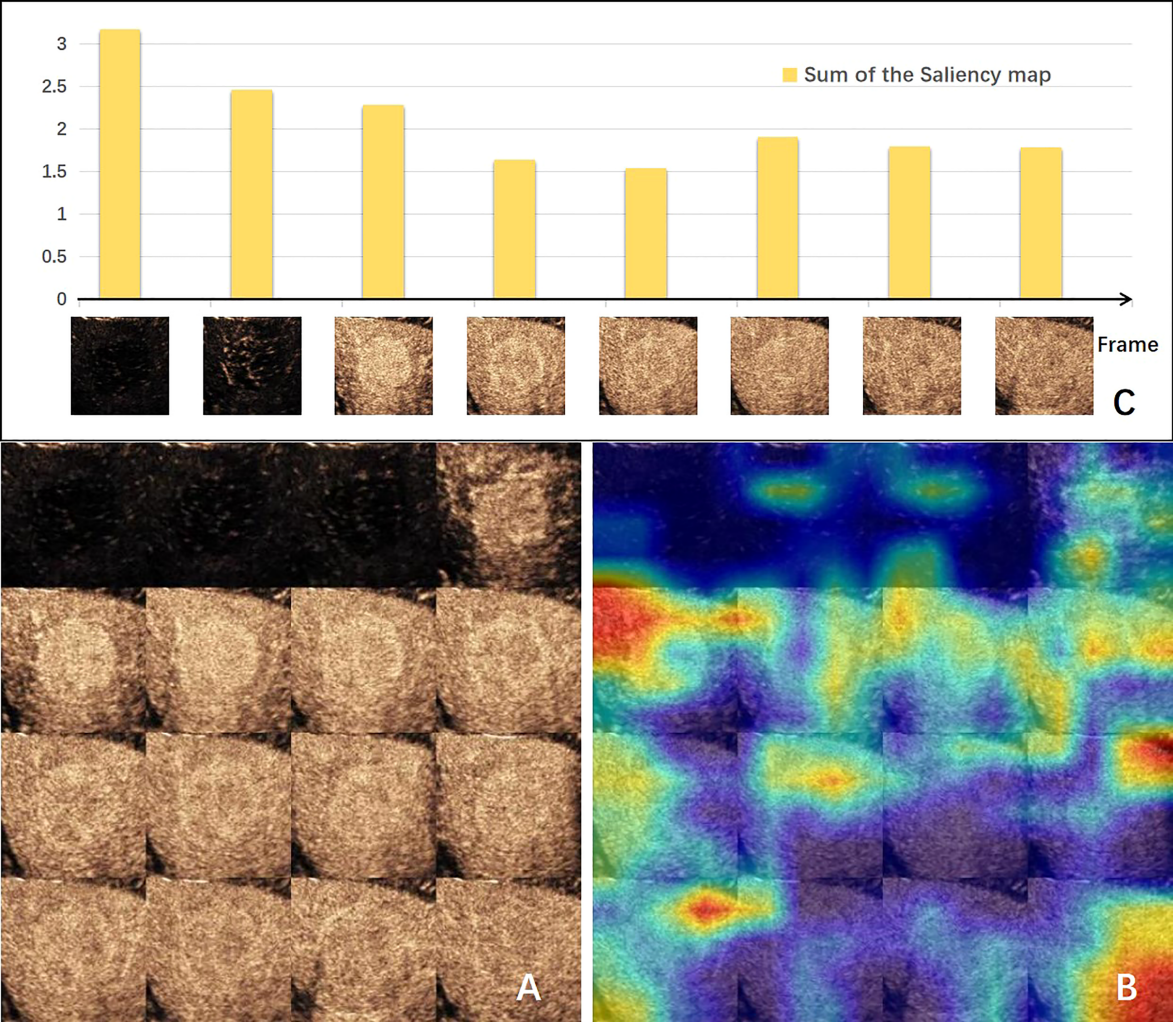
Visual explanation of the deep convolution neural network (DCNN) model. **(A)** Input of CNN-based module made by 16-frame sliced images extracted from the 1-min video. **(B)** Corresponding gradient-weighted class activation map. Highlighted areas were the network paid attention for MVI prediction. **(C)** Bar chart of the sum of the saliency maps of each input frame for the GRU-based module. The value indicates the degree of the importance for this frame predicting MVI. CNN, convolution neural network; MVI, microvascular invasion.

The visual explanation of the spatial–temporal features extracted by the DCNN model is shown in **Figure 3**. The areas that the CNN module paid most attention to were the periphery and neighboring of the tumor, conforming to the regions where MVI probably existed (**Figures 3A, B**). Generally, the early arterial phase of contrast agent perfusion into the liver is the most sensitive period for visualization of the vascular bed. The first two bars in **Figure 3C**, representing the pre-arterial phase and the beginning of the arterial phase, were the tallest of all, which means they made the most contributions to the prediction of MVI status. The contributions of the frames declined afterwards, but got high at the sixth frame when the arterial phase alternated with the portal vein phase, which is important for diagnosis. These changes in the frames’ contributions produced by the GRU-based module were consistent with the clinical experience.

**Table 3** summarizes the predictive performances of the three models. Compared with the Clinical model, the CEUS-DCNN model exhibited similar sensitivity, but higher specificity (76.1% vs. 63.4%, *p* = 0.05 in the validation group; 71.4% vs. 38.1%, *p* = 0.03 in the test group). The CECL-DCNN model, which incorporated CEUS video and clinical parameters, achieved not only higher specificity (86.6% vs. 63.4%, *p <* 0.001 in the validation group; 81.0% vs. 38.1%, *p* = 0.005 in the test group) but also higher accuracy than the Clinical model (81.4% vs. 68.0%, *p* = 0.008 in the validation group; 78.8% vs. 51.5%, *p* = 0.009 in the test group). Additionally, the CECL-DCNN model had a promising diagnostic performance (AUC = 0.879 in the validation group and AUC = 0.865 in the test group).

**Table 3:** Predictive efficacy of the clinical, CEUS-DCNN, and CECL-DCNN models.

Model		Sensitivity	Specificity	Accuracy	AUC
**Clinical model**	Validation*	76.8%	63.4%	68.0%	0.765
	Test	75.0%	38.1%	51.5%	0.732
**CEUS-DCNN model**	Validation	71.4%	76.1% ^†^	74.5%	0.832
	Test	75.0%	71.4% ^†^	72.7%	0.734
**CECL-DCNN model**	Validation	71.4%	86.6% ^†^	81.4% ^†^	0.879 ^†^
	Test	83.3%	81.0% ^†^	78.8% ^†^	0.865

AUC, area under the curve. *Multivariate logistic analysis was used. ^†^The comparison with the clinical model was significant, p < 0.05.

### Long-Term Prognosis of Predicted MVI

The study was censored on September 30, 2020. The median follow-up was 63.6 months (interquartile range, 46.2–77.5) in all 436 patients. The mean RFS was 37.2 months (95% confidence interval [CI]: 30.6–43.9) for those with histologic MVI and 63.5 months (95% CI: 58.6–68.5) for those without histologic MVI (*p* < 0.001). The mean OS was 62.3 months (95% CI: 22.9–68.7) for those with histologic MVI and 88.9 months (95% CI: 85.5–92.4) for those without histologic MVI (*p* < 0.001). Histologic MVI was confirmed to be an important prognostic factor for poor prognosis.

**Figure 4:**
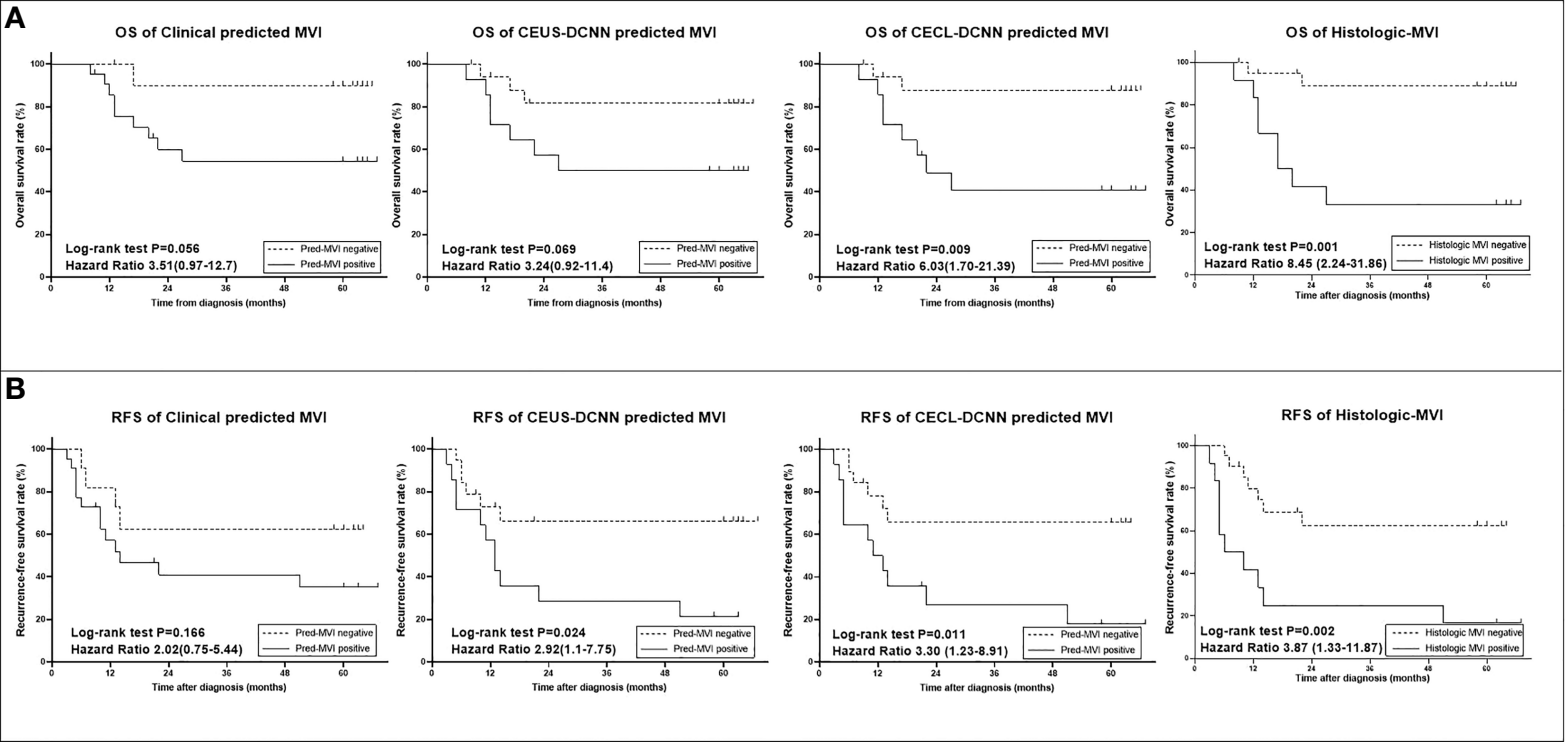
Survival curves of histologic microvascular invasion (MVI) and predicted MVI of the three models in the test group (*n* = 33). **(A)** Overall survival (OS) curves. **(B)** Recurrence-free survival (RFS) curves. Comparisons between curves were performed with the log-rank test. CEUS-DCNN: CEUS video-based deep convolution neural network model. CECL-DCNN: clinical parameter combining CEUS-based deep convolution neural network model.

The predicted MVI of the three models could significantly distinguish the patients with poor outcomes in the validation group (*p* < 0.05, seen in **Supplementary Figure 2**), but in the test group, the prognostic results of the three models were different (**Figure 4**). The prognostic effects of the Clinical model predicted MVI on survival and recurrence did not reach any statistical significance (both *p* > 0.05), while in the same group, the CEUS-DCNN predicted MVI was a significant prognostic factor only for RFS (hazard ratio [HR] with 95% CI: 2.92 [1.1–7.75], *p* = 0.024). Nevertheless, the CECL-DCNN predicted MVI could significantly distinguish the patients with shorter survival (mean OS = 37.3 vs. 59.7 months, HR with 95% CI: 6.03 [1.7–21.39], *p* = 0.009) and earlier recurrence (mean RFS = 23.5 vs. 45.3 months, HR with 95% CI: 3.3 [1.23–8.91], *p* = 0.011). The survival curves of the CECL-DCNN predicted MVI were the most similar to that of the histologic MVI (**Figure 4**). The patients with positive MVI had shorter survival (mean OS = 32.9 vs. 60.5 months, HR with 95% CI: 8.45 [2.24–31.86], *p* = 0.001) and earlier recurrence (mean RFS = 20.8 vs. 44.6 months, HR with 95% CI: 3.87 [1.32–11.31], *p* = 0.002).

## Discussion

In the present study, we proposed a CECL-DCNN model, which integrated clinical information and temporal–spatial information from the CEUS video, to predict histologic MVI in patients with HCC. The CECL-DCNN model showed significantly higher specificity and accuracy than the Clinical model in validation and test groups, achieving a satisfying diagnostic performance (AUC = 0.879 and 0.865 in two groups, respectively). Additionally, the CECL-DCNN predicted MVI was a prognostic factor to poor long-term outcomes, indicating its impact on clinical decisions before surgery.

Compared to CT and MR, US has the advantages of being readily accessible, radiation-free, and easy to operate and having economic benefits. Furthermore, CEUS allows real-time evaluation of the enhancement of a nodule, providing more sensitive detection of arterial phase enhancement (APHE) than CT or MR, which may fail to demonstrate APHE due to the arterial phase mistiming (23). Previously, Zhang et al. found that the CEUS radiomics nomogram could predict MVI with an AUC of 0.788 in the validation dataset, but the specificity was only 70.83% (24). Zhou et al. found that CEUS LR-M combining clinical features could predict MVI with an AUC of 0.84, but the specificity was slightly lower than the clinical model (78.6% vs. 85.7%, *p* = 0.06) (25). In this study, the CEUS-DCNN model, which only used CEUS information, could predict MVI with a significantly higher specificity than the Clinical model. As regards clinical information, the CECL-DCNN model could achieve better specificity (86.6% in the validation group and 81.0% in the test group) and accuracy (81.4% in the validation group and 78.8% in the test group). The specificity of our study was much higher than the results of the above two CEUS studies. Xu et al. (12) reported that radiomics of enhanced CT predicted MVI with an AUC of 0.889 and a specificity of 79.2%, and Yang et al. (26) found that enhanced MR could predict MVI with an AUC of 0.861 and a specificity of 81.4%. Our result was as good as theirs. Furthermore, many studies lacked survival analysis to validate the model’s validity. In this study, the CECL-DCNN predicted MVI demonstrated the most similarity to true histologic MVI of the three models in the comparison of survival curves, indicating the reliability of this model. Accordingly, our study provided another straightforward, noninvasive, and robust approach for predicting MVI before surgery.

Deep learning is a state-of-the-art machine learning approach. Early studies of deep learning applied to MVI prediction based on enhanced CT or MR have reported superior performances (27, 28), but they did not evaluate the model in an independent group. Liu et al. (29) found that deep learning of enhanced CT could predict MVI with an AUC of 0.777, and Wei et al. (30) reported that deep learning of enhanced MR and enhanced CT could predict MVI with an AUC of 0.812 and 0.736, respectively, in the external test group. Our study established an independent test group in addition to the training and validation groups, and the deep learning model based on CEUS and clinical variables made excellent diagnostic performances with an AUC of 0.865 in the test groups.

At present, there are no studies about deep learning of CEUS to predict MVI. This is probably because CEUS was a dynamic video with a high spatial and temporal complexity, and quantitative analysis of CEUS is difficult. Previously, Xie and Tian’s team found that a deep learning radiomics-based CEUS model could accurately predict the response to TACE for HCC patients (31) and could predict prognosis literally after surgery and radiofrequency ablation to help patients with treatment decision-making (32), which inspired us to use deep learning to analyze CEUS video. Compared to the traditional radiomics method, our DCNN model did not pre-define features in terms of feature selection and extraction. Moreover, DCNN algorithms have great advantages at learning features in a data-driven mode and thus make predictions more practical (33). In this study, we developed a DCNN model made up of the GRU-based module and the CNN-based module, focusing on temporal information and texture information, respectively. Considering the computing cost and redundant information in CEUS video, frames were uniformly sampled from videos, and short-time and long-time intervals were used separately when forming the inputs of the CNN-based and GRU-based modules. Inputs of the CNN- and GRU-based modules included frames in both the arterial phase and the portal phase, and thus, our model could thoroughly use the information in the video.

It should be noted that this study has some limitations. On the one hand, this was a single-center retrospective study. Therefore, results from our center should be supplemented with further prospective validation by larger cohorts from other centers. On the other hand, although manually segmenting the tumor is relatively precise, it is tedious and laborious. Next, we will develop an algorithm for automatic video object segmentation.

## Conclusion

The proposed CECL-DCNN model, based on preoperative CEUS video and clinical parameters, can serve as a noninvasive tool to predict MVI status in HCC, thereby predicting poor long-term outcomes, indicating its impact on clinical decisions before the surgery.

## Data Availability Statement

The raw data supporting the conclusions of this article will be made available by the authors, without undue reservation.

## Ethics Statement

The studies involving human participants were reviewed and approved by The ethics committee of Sun Yat-Sen University Cancer Center. The ethics committee waived the requirement of written informed consent for participation.

## Author Contributions

Conception and design: JZ and JY. Development of methodology: QW and YZ. Acquisition of data (acquired and managed patients and prognosis, and acquired CEUS): YZ, CY, YH, and XZ. Data analysis and interpretation (ROI segmentation, computational analysis, and statistical analysis): YZ and QW. Writing, review, and/or revision of the manuscript: YZ, QW, JZ, and JW. Administrative, technical, technical, or material support: YZ and QW. Study supervision and guarantors: JZ and JY. All authors contributed to the article and approved the submitted version.

## Conflict of Interest

The authors declare that the research was conducted in the absence of any commercial or financial relationships that could be construed as a potential conflict of interest.

## Publisher’s Note

All claims expressed in this article are solely those of the authors and do not necessarily represent those of their affiliated organizations, or those of the publisher, the editors and the reviewers. Any product that may be evaluated in this article, or claim that may be made by its manufacturer, is not guaranteed or endorsed by the publisher.

## Supplementary Material

Supplementary Figure 1 The region of interest (ROI) segmentation in CEUS. **(A)**An example of annotations manually made in each frame of one CEUS cine (Red line). **(B)** A mask was formed from the union of annotations based on each frames’ annotations projection in time dimension. **(C)** Annotation manually drawn (Red line). A bounding box according to the union of annotations (Green line). Extension of the bounding box (Blue line).

Supplementary Figure 2 Survival curves of histologic microvascular invasion (MVI) and predicted MVI of the three models in validation group (n=102). **(A)** Overall survival (OS) curves. **(B)** Recurrence-free survival (RFS) curves. Comparisons between curves were performed with the log-rank test. CEUS-DCNN: CEUS video- based deep convolution neural network model. CECL-DCNN: clinical parameter combining CEUS-based deep convolution neural network model.
