# Supplementary figures and images for: Deep Learning of Liver Contrast-Enhanced Ultrasound to Predict Microvascular Invasion and Prognosis in Hepatocellular Carcinoma
Source: Front Oncol. 2022 Jul 7;12:878061. doi: 10.3389/fonc.2022.878061 (PMC9300962; doi:10.3389/fonc.2022.878061)

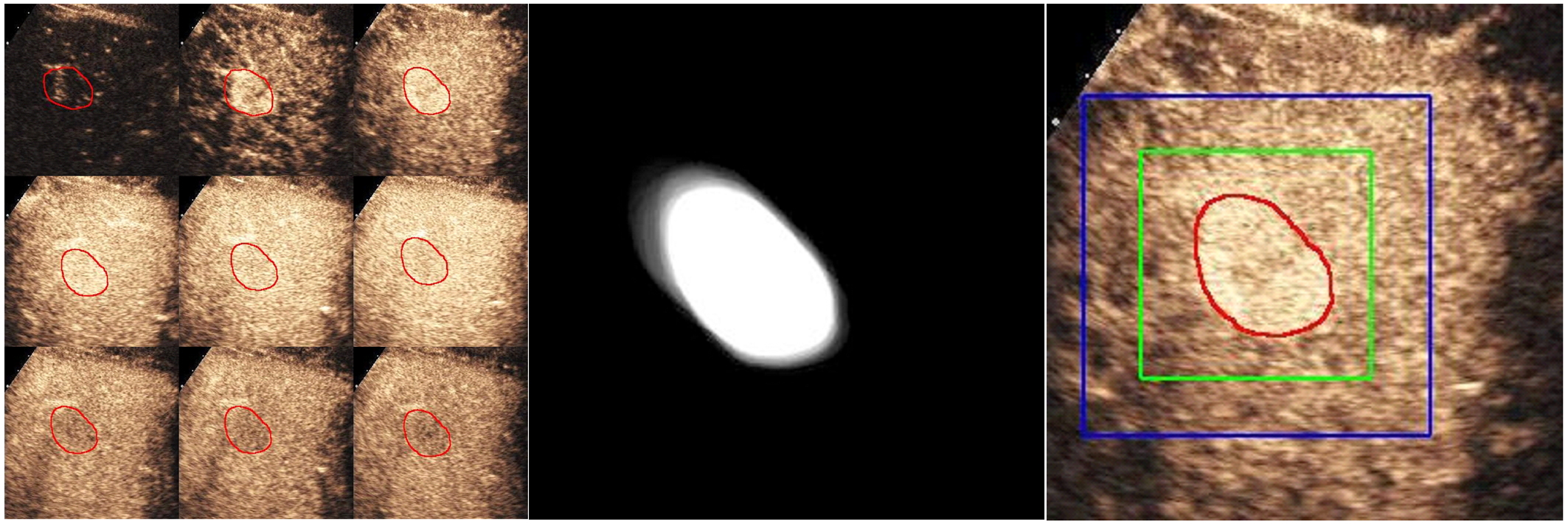

Supplement: Supplementary Figure 1 — The region of interest (ROI) segmentation in CEUS. (A)An example of annotations manually made in each frame of one CEUS cine (Red line). (B) A mask was formed from the union of annotations based on each frames’ annotations projection in time dimension. (C) Annotation manually drawn (Red line). A bounding box according to the union of annotations (Green line). Extension of the bounding box (Blue line). [file Image_1.jpeg]

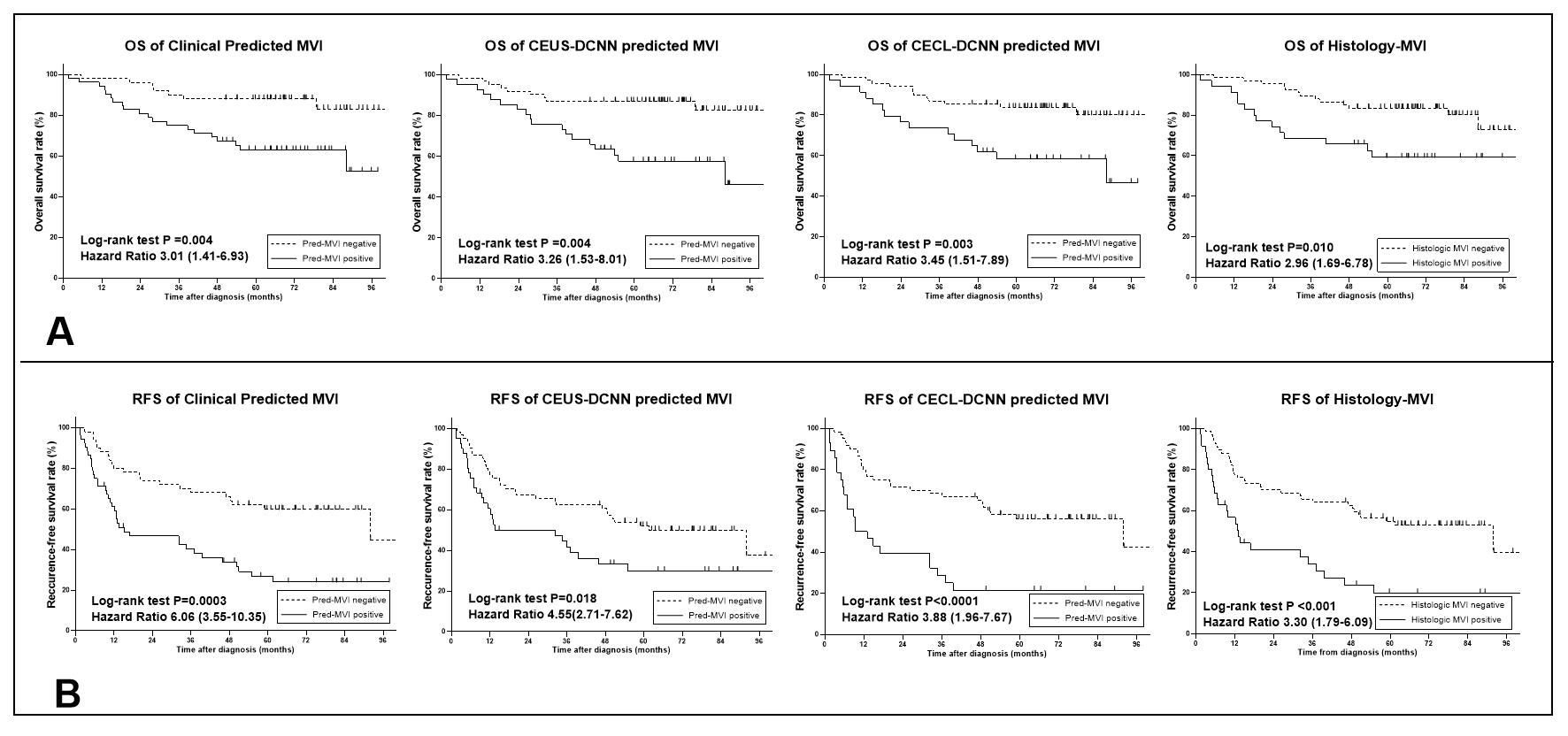

Supplement: Supplementary Figure 2 — Survival curves of histologic microvascular invasion (MVI) and predicted MVI of the three models in validation group (n=102). (A) Overall survival (OS) curves. (B) Recurrence-free survival (RFS) curves. Comparisons between curves were performed with the log-rank test. CEUS-DCNN: CEUS video- based deep convolution neural network model. CECL-DCNN: clinical parameter combining CEUS-based deep convolution neural network model. [file Image_2.jpg]
