# Supplementary material for: Deep Learning of Liver Contrast-Enhanced Ultrasound to Predict Microvascular Invasion and Prognosis in Hepatocellular Carcinoma
Source: Front Oncol. 2022 Jul 7;12:878061. doi: 10.3389/fonc.2022.878061 (PMC9300962; doi:10.3389/fonc.2022.878061)
Supplement: Supplementary file 3 [file DataSheet_1.docx]

Network Architecture

The proposed model consists of two parts: a Gated Recurrent Unit (GRU) based module and a Convolution neural networks (CNN)-based module.

GRU-based module was seamlessly composed by Inception V4 as backbone and two-stage cascade Bidirectional GRU [1]. This module aimed to focus on the temporal pattern of CEUS videos. InceptionV4 was pre-trained on ImageNet. Let $F_{Incep}$be the output feature of InceptionV4, $F_{Incep}\in R^{N\times W}$, where $N$ was the length of the input frame-sequence and $W=1536$. $F_{Incep}$then was taken as input by the two-stage GRU. Denote the output of the first stage GRU as $\mathrm{Output}_{GRU1}\in R^{N\times L_{1}}$, where $L_{1}$ was the length of cell’s output in stage one. Input of the second stage GRU was a concatenation of every two cells’ outputs in stage one. Output of the first cell in stage two was the final output of the GRU-based module, denoted as $F_{G}\in R^{L_{2}}$, where $L_{2}$ was the length of cell’s output in stage two.

CNN-based module was developed based on ResNet50. ResNet50 was used as a feature extractor in this module, denoted as $E$. Three different scales of extracted feature map from ResNet50 was denoted as $F_{Res} \in R^{{C_{s}\times H}_{s}\times W_{s}}, s=\{1, 2, 3\}$, where $C_{s}, H_{s}{, W}_{s}$ were the channel number, height and width of the feature map in $s$ stage. And channel numbers were set to be 512, 1024, 2048 respectively. Selected feature map then separately became the input of the Conv Block from corresponding pipelines, denoted as $H_{CB}^{i}, i=\{1, 2, 3\}$ which contained two cascade pattern of Convolution, Batch Normalization[2], and Elu[3] (**Supplementary Fig.3**, detailed parameters shown in **Supplementary Tab.1**).


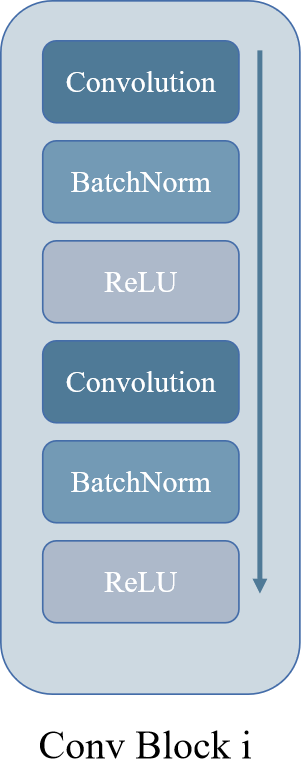


**Supplementary Figure 3. Structure of Conv Block, i= {1,2,3}.**

**Supplementary Table 1. Detailed network parameters of Conv Block i, i = {1, 2, 3}**

| **Layer name** | **i = 1** | **i = 2** | **i = 3** |
| --- | --- | --- | --- |
|  | Input size | | |
|  | 512$\times$69$\times$69 | 1024$\times$35$\times$35 | 2048$\times$18$\times$18 |
| **Conv_1** | 512, 512  1$\times$1, stride 1, padding 0 | 1024, 512  1$\times$1, stride 1, padding 0 | 2048, 512  1$\times$1, stride 1, padding 0 |
| **bn_1** | 512  eps=1e-5, momentum=0.01 | 512  eps=1e-5, momentum=0.01, | 512  eps=1e-5, momentum=0.01 |
| **relu_1** | | | |
| **Conv_2** | 512, 1024  3$\times$3, stride 1, padding 1 | 512, 1024  3$\times$3, stride 1, padding 1 | 512, 1024  3$\times$3, stride 1, padding 1 |
| **bn_2** | 1024  eps=1e-5, momentum=0.01 | 1024  eps=1e-5, momentum=0.01 | 1024  eps=1e-5, momentum=0.01 |
| **relu_2** | | | |

The Convolution layer from the first stage cascade pattern of Conv Blocks in the three pipelines had the different channel number which is consistent with its corresponding input feature map. Other layers in the three pipelines had the same parameter settings. Denote $F_{H}^{i}\in R^{C\times H_{0}^{i}\times W_{0}^{i}}$ as the output of $H_{CB}^{i}$, and here we set $C=1024$. Max Pooling was then used to reduce the height and width of $F_{H}^{i}$ into 1. Denoted $F_{M}^{i}\in R^{C\times1\times1}$ as the output of Max Pooling. Then reshaped $F_{M}^{i}\in R^{C\times1\times1}$ to ${F'}_{M}^{i}\in R^{C}$. Thereafter, FC Block which had two cascade fully-connected (FC) layers presented as $H_{FC}^{i}, i=\{1, 2, 3\}$was adopted for prediction as $y_{pred}^{i}=H_{FC}^{i}\left( {F^{'}}_{M}^{i} \right)$ (**Supplementary Fig.4**, detailed parameters shown in **Supplementary Tab.2** )


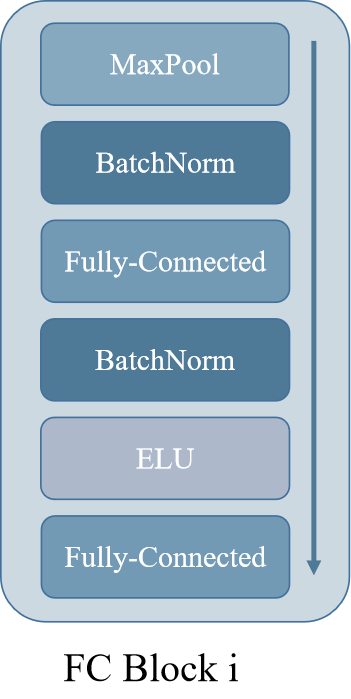


**Supplementary Figure 4. Structure of fully-connected (FC) Block, i= {1,2,3}**

**Supplementary Table 2 Detailed network parameters of fully-connected (FC) Block i, i = {1, 2, 3}**

| **Layer name** | **i = 1** | **i = 2** | **i = 3** |
| --- | --- | --- | --- |
|  | Input size | | |
|  | 1024$\times$69$\times$69 | 1024$\times$35$\times$35 | 1024$\times$18$\times$18 |
| **Max Pool** | kernel 56  stride 56 | kernel 28  stride 28 | kernel 14  stride14 |
| **bn_1** | 1024  eps=1e-5, momentum=0.1 | 1024  eps=1e-5, momentum=0.1 | 1024  eps=1e-5, momentum=0.1 |
| **linear_1** | 1024, 512 | 1024, 512 | 1024, 512 |
| **bn_2** | 512  eps=1e-5, momentum=0.1 | 512  eps=1e-5, momentum=0.1 | 512  eps=1e-5, momentum=0.1 |
| **Elu** | alpha=1.0 | alpha=1.0 | alpha=1.0 |
| **linear_2** | 512, 30 | 512, 30 | 512, 30 |

We also added a three-stage FC layers to make predictions according to feature obtained in three different pip-lines, GRU-based module and clinical variables. Denoted these three FC layers separately as FC Block 4 ${(H}_{FC1}^{concat})$, FC Block 5 $(H_{FC2}^{concat})$ and FC 4$(H_{FC3}^{concat})$. The detailed network structure of FC Block 4, 5 are shown in **Supplementary Table 3.** Firstly, concatenate ${F'}_{M}^{i}, i=\{1, 2, 3\}$ as ${F'}_{M}^{concat}$. The output of $H_{FC1}^{concat}$ could be obtained as $F_{FC1}^{concat}=H_{FC1}^{concat}({F'}_{M}^{concat}), F_{FC1}^{concat}\in R^{D_{1}}$, where $D_{1}$ was the feature size of the first FC layer’s output. By concatenating $F_{FC1}^{concat}$ and output of the GRU-based module $F_{G}$, we could have the input of $H_{FC2}^{concat}$. And its output was defined as $F_{FC2}^{concat}, F_{FC2}^{concat}\in R^{D_{2}}$.The input of last FC layer was concatenated by $F_{FC2}^{concat}$ and $F_{clinic}$. Thus, $y_{pred}^{concat}$ could be defined as$y_{pred}^{concat}=H_{FC3}^{concat}(concat[F_{clinic},H_{FC2}^{concat}\left( concat[{F_{G}, H}_{FC1}^{concat}\left( concat[{F^{'}}_{M}^{1}, {F^{'}}_{M}^{2}, {F^{'}}_{M}^{3}] \right)] \right)])$.

In the proposed module, we set $D_{1}=512, D_{2}=30$. And the final prediction was $y_{pred}^{concat}$.

**Supplementary Table 3 Detailed network parameters of fully-connected (FC) Block i, i = {4, 5}**

| **Layer name** | **i = 4** | **i = 5** |
| --- | --- | --- |
|  | Input size | |
|  | 3072 | 640 |
| **bn_1** | 3072  eps=1e-5,  momentum=0.1 | / |
| **linear_1** | 3072, 512 | 640, 30 |
| **bn_2** | 512  eps=1e-5,  momentum=0.1 | / |
| **Elu** | alpha=1.0 | / |

Training strategy

Inspired by the Progressive Multi-Granularity (PMG) training framework[4], a progressive training strategy was adopted in the experiment. We firstly trained the low stage pipeline where its receptive field and representation ability was limited. The low stage pipeline had the smallest input feature map size. Then the higher stages were progressively trained. In this strategy, the proposed model could gradually learn features from local to global.

During training, a jigsaw puzzle generator[4] was also applied for the input images of CNN-based module. When training different pipelines of CNN-based module, the size of exchanging patches would also be different. Here, for pipelines whose input feature’s channel numbers were 512, 1024, 2048, their corresponding patch sizes were set as 1/10, 1/4, 1/2 of the original image sizes separately. The input for the CNN-based module based on the concatenation features was the original image.

This strategy forced our model to find more discriminative parts, because the generated images had different and variable receptive fields. And it increased the robustness of the proposed model since it could be seen as a special strategy for data augmentation. In our training step, we adopted the focal loss[5] to prevent the vast number of easy negatives from overwhelming the classifier during training, since negative samples were the majority in our collected data. While training, we set learning rate at 0.002 and applied the SGD optimizer to optimize the model parameters

**Reference**

1. Gulcehre C, Cho K, Pascanu R, Bengio Y. Learned-Norm Pooling for Deep Feedforward and Recurrent Neural Networks. Joint European Conference on Machine Learning and Knowledge Discovery in Databases. 2014.

2. Ioffe S, Szegedy C. Batch normalization: accelerating deep network training by reducing internal covariate shift. Proceedings of the 32nd International Conference on International Conference on Machine Learning - Volume 37. Lille, France: JMLR.org; 2015. p. 448–56.

3. Clevert D-A, Unterthiner T, Hochreiter S. Fast and Accurate Deep Network Learning by Exponential Linear Units (ELUs). arXiv: Learning. 2016.

4. Ruoyi Du DC, Ayan Kumar Bhunia, Jiyang Xie, Zhanyu Ma, Yi-Zhe Song, Jun Guo. Fine-Grained Visual Classification via Progressive Multi-Granularity Training of Jigsaw Patches. Computer Vision and Pattern Recognition. 2020.

5. Lin TY, Goyal P, Girshick R, He K, Dollar P. Focal Loss for Dense Object Detection. 2017 IEEE International Conference on Computer Vision (ICCV)2017.
